# Omega-7 oil increases telomerase activity and accelerates healing of grafted burn and donor site wounds

**DOI:** 10.1038/s41598-020-79597-0

**Published:** 2021-01-13

**Authors:** Yosuke Niimi, Dannelys Pérez-Bello, Koji Ihara, Satoshi Fukuda, Sam Jacob, Clark R. Andersen, Tuvshintugs Baljinnyam, Jisoo Kim, Suzan Alharbi, Donald S. Prough, Perenlei Enkhbaatar

**Affiliations:** 1 Department of Anesthesiology, Medical Branch, University of Texas, 301 University Blvd, Galveston, TX 77555-1102 USA; 2 Department of Plastic and Reconstructive Surgery, Tokyo Women’s Medical University, 8-1, Kawada-cho, Shinjuku, Tokyo Japan; 3 Department of Pathology, Shriners Hospitals for Children, 815 Market St, Galveston, TX 77550 USA; 4 Department of Biostatistics, Medical Branch, University of Texas, 301 University Blvd, Galveston, TX 77555-1102 USA

**Keywords:** Drug discovery, Molecular biology, Plant sciences, Diseases

## Abstract

This study investigated the efficacy of Omega-7 isolated from the sea buckthorn oil (Polyvit Co., Ltd, Gangar Holding, Ulaanbaatar, Mongolia) in ovine burn wound healing models. In vitro, proliferation (colony-forming rate) and migration (scratch) assays using cultured primary ovine keratinocytes were performed with or without 0.025% and 0.08% Omega-7, respectively. The colony-forming rate of keratinocytes in the Omega-7 group at 72 and 96 h were significantly higher than in the control (P < 0.05). The percentage of closure in scratch assay in the Omega-7 group was significantly higher than in the control at 17 h (P < 0.05). In vivo, efficacy of 4% Omega-7 isolated from buckthorn oil was assessed at 7 and 14 days in grafted ovine burn and donor site wounds. Telomerase activity, keratinocyte growth factor, and wound nitrotyrosine levels were measured at day 14. Grafted sites: Un-epithelialized raw surface area was significantly lower and blood flow was significantly higher in the Omega-7-treated sites than in control sites at 7 and 14 days (P < 0.05). Telomerase activity and levels of keratinocyte growth factors were significantly higher in the Omega-7-treated sites after 14 days compared to those of control (P < 0.05). The wound 3-nitrotyrosine levels were significantly reduced by Omega-7. Donor sites: the complete epithelialization time was significantly shorter and blood flow at day 7 was significantly higher in the Omega-7-treated sites compared to control sites (P < 0.05). In summary, topical application of Omega-7 accelerates healing of both grafted burn and donor site wounds. Omega-7 should be considered as a cost-efficient and effective supplement therapy for burn wound healing.

## Introduction

In the U.S., 486,000 burn injuries requiring medical treatment occur annually^1^. The average daily hospital cost of surviving patients in the U.S., is $8179^2^. Delayed wound healing of severely burned patients and limited availability of donor skin remain challenging problems^3^. Thus, an efficient and robust therapy for healing of both grafted burn and donor sites is needed.

The wound healing process is dynamic and has three overlapping phases (i.e., inflammation, tissue formation, and tissue remodeling) that occur in a specific time and duration^4^. Optimal bio-physiologic events of the healing process involve the following: appropriate inflammation; angiogenesis; re-epithelialization; mesenchymal cell differentiation, proliferation, and migration; and collagen remodeling—cross-linking and strength^5^. These wound healing events are affected by local factors, such as cytokines (i.e., keratinocyte growth factor, vascular endothelial growth factor, fibroblast growth factor, transforming growth factor beta, and interleukin-1), blood cells, extracellular matrix, and parenchymal cells.

Telomeres and telomerases play essential roles in the control of cell proliferation. Telomerase is a ribonucleoprotein complex that extends telomeres. It is known that telomerase maintains telomere length in various cells^6^. When cells divide, telomere length is shortened with each round of DNA replication leading to senescence^7,8^.

Skin cell telomeres may be particularly susceptible to accelerated shortening due to excess generation of reactive oxygen species (ROS) in burn wounds which damage cellular DNA and negatively affect proliferation^9,10^. The healthy length of telomeres is maintained by a number of factors, including telomerase^11^. Although telomerase is known to be activated in the epidermis and it plays a significant role in the maintenance of skin cell functions and proliferation, it is not known if telomerase activity is negatively affected by burns^12^.

Various treatment approaches have been proposed to improve healing of both grafted burn and donor site wounds, including topical medications and tissue engineered materials (i.e., fibroblast growth factor^13^, artificial dermis templates^14^, cultured epithelial allograft^15^, and/or cellular therapy such as mesenchymal stem cells^16^, induced pluripotent stem cells^17^, and embryonic stem cells^18^). However, high cost, long culture time, and ethical issues often prevent their use in clinical practice.

There are several reports on the use of natural products, such as *Allium sativum*, Aloe vera, and *Centella asiatica* for treatment of burn wounds. Previously, we have reported the effects of sea buckthorn (*Hippophae rhamnoides* L.) oil on grafted burn wound healing^19–21^. Sea buckthorn is a wild berry of the *Elaeagnaceae* family growing at an elevation of 2500–4300 m in Europe and Asia^22^. The plant has been used extensively in traditional medicine in Asia for treating burn wounds for more than 1000 years^23^. Omega-7 unsaturated oils constitute approximately 48% of sea buckthorn oil^21^. Although sea buckthorn oil has been shown to promote skin and mucosal epithelialization^24,25^, effects of Omega-7 in wound healing are not known. In the present study, we have tested the hypothesis that Omega-7 improves healing of both grafted burn and donor site wounds.

## Results

### Number of colonies in cultured keratinocytes and keratinocyte wound closure rate in vitro

**Figure 1:**
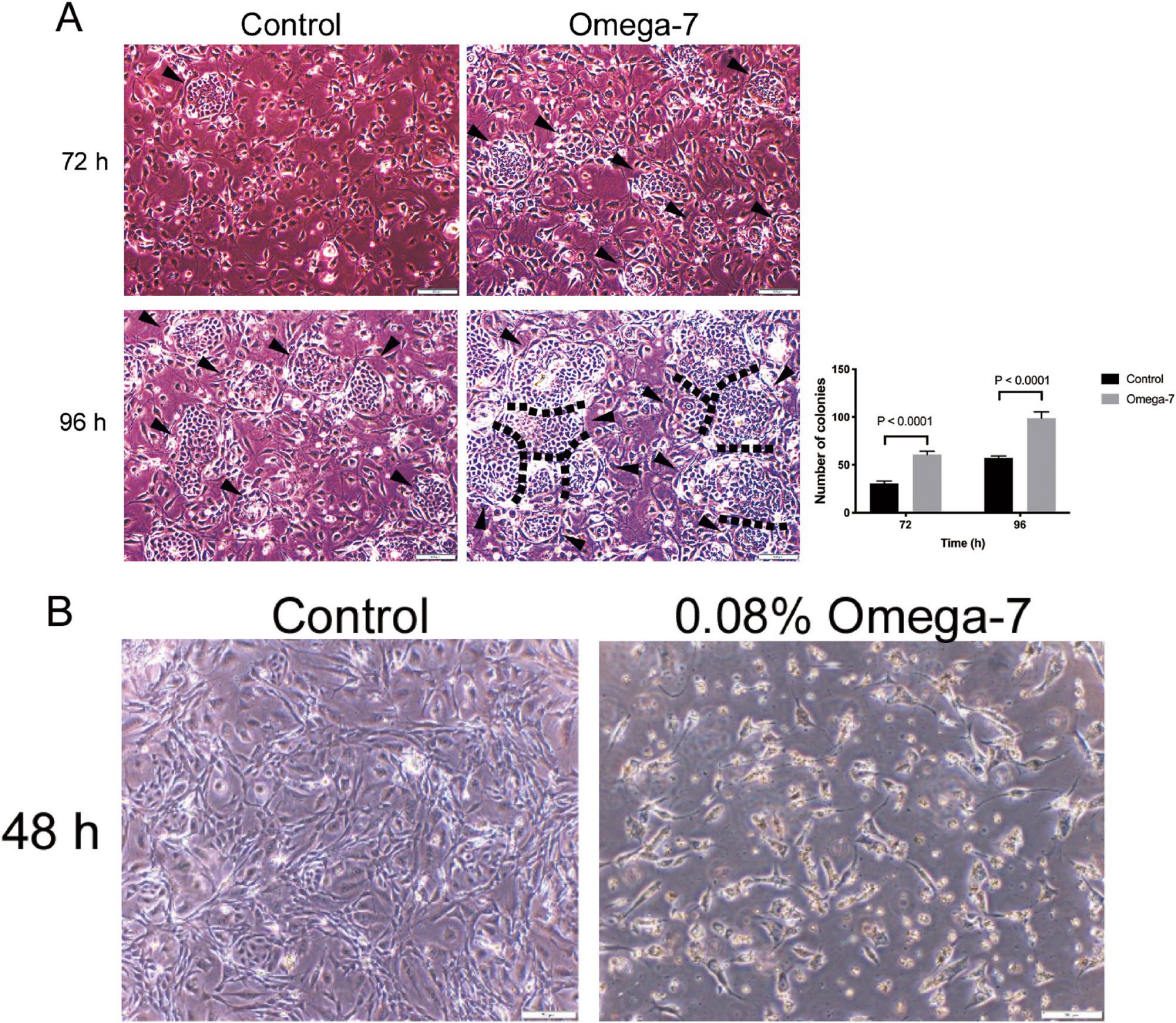
Effects of Omega-7 on the cultured keratinocyte colony-forming rate. (**A**) Effects of 0.025% omega-7 on cultured keratinocyte colony-forming rate (proliferation) at 72 and 96 h. (**B**) Effects of 0.08% of Omega-7 on cultured keratinocyte colony-forming rate at 48 h. Omega-7 with dose of 0.08% negatively affected the cells. Keratinocyte colony counting assay was performed using 24,000 cells/cm^2^ sheep primary keratinocytes in passage 2. Cells were treated with Omega-7 at time 0 h. The colonies were counted in 12 dishes (6 treated and 6 controls). Data are expressed as mean ± SEM. Scale bar in micrographs: 200 μm. Open circles indicate cells treated with Omega-7 and closed circles represent control cells. Photographs taken and compiled, by authors, in Adobe Photoshop CC 2020 (https://www.adobe.com/jp/products/photoshop.html) without changing the content of images themselves.

The proliferation and migration rates of cultured keratinocytes were assessed by the colony counting and scratch assays, respectively. The number of keratinocyte colonies in the Omega-7 group treated with 0.025% of Omega-7 at 72 and 96 h (60.8 ± 3.3, and 98.8 ± 6.8, respectively) was significantly higher than that of the control group (30.5 ± 2.8, and 57.4 ± 1.9, respectively) (P < 0.0001) (Fig. 1A). Omega-7 with dose of 0.08% negatively affected the cells (Fig. 1B).

**Figure 2:**
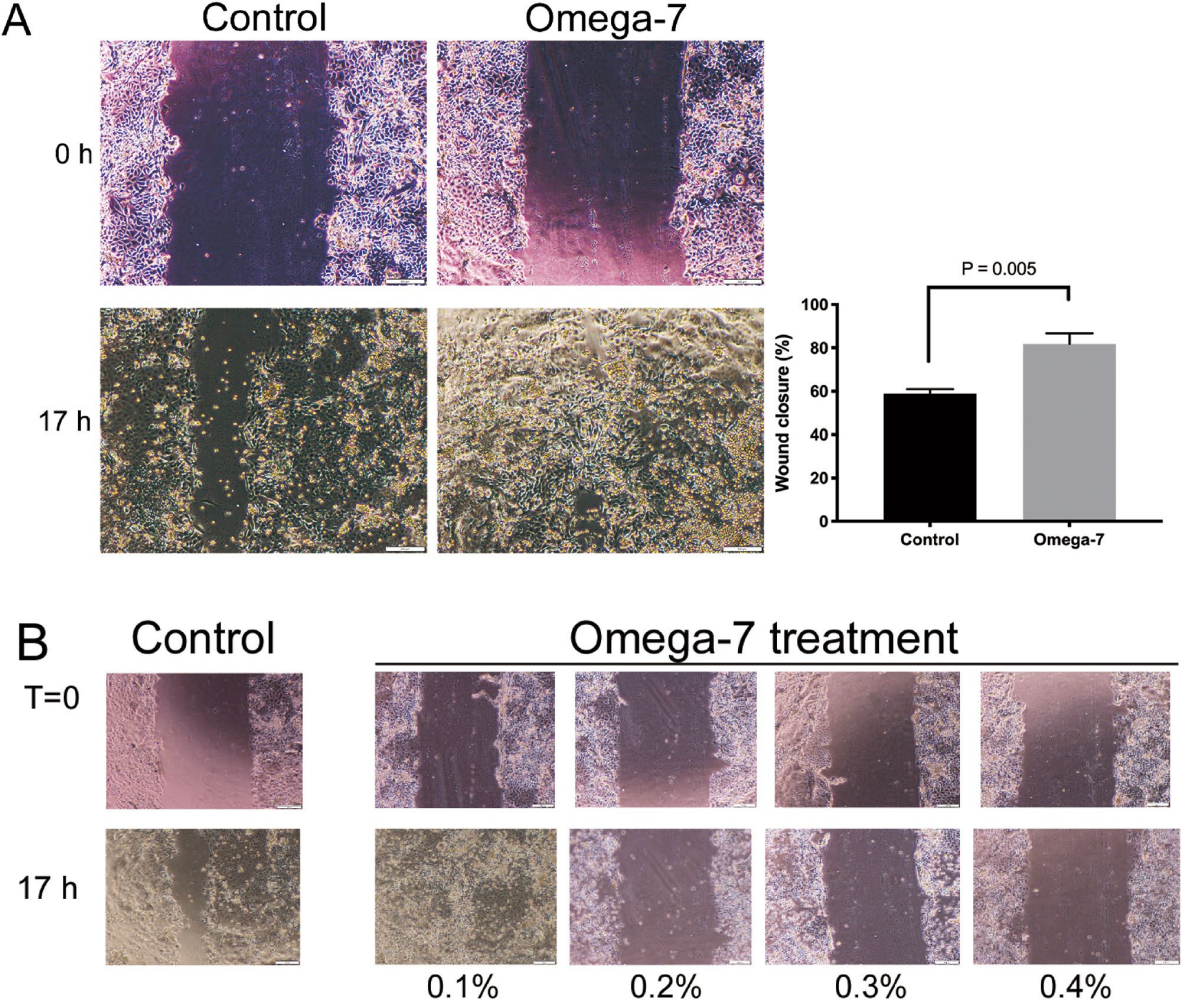
Cultured keratinocyte migration assay. (**A**) Effects of 0.08% Omega-7 on the cultured keratinocyte migration rate 17 h after the scratch. (**B**) Effects of Omega-7 (0.1, 0.2, 0.3, and 0.4%) on cultured keratinocyte migration rate. The scratch was performed when the cells were grown confluently. The Omega-7 was added to the confluently grown cells immediately after the scratch. Omega-7 had a negative effect on the keratinocyte migration with dose greater than 0.1%. The cells were assessed in 12 dishes (6 treated and 6 controls). Data are expressed as mean ± SEM. Scale bar in micrographs: 200 μm. Photographs taken and compiled, by authors, in Adobe Photoshop CC 2020 (https://www.adobe.com/jp/products/photoshop.html) without changing the content of images themselves.

The percentage of cultured keratinocyte wound closure (scratch assay) in the Omega-7 group at 17 h after scratch (81.8 ± 4.9) was significantly higher than that of the control group (58.9 ± 2) (P = 0.005) (Fig. 2A). Omega-7 with doses of greater than 0.1% negatively affected the cell migration (Fig. 2B).

### Un-epithelialized raw surface area in grafted burn wounds and time (number of days) of complete re-epithelialization of donor sites in vivo

**Figure 3:**
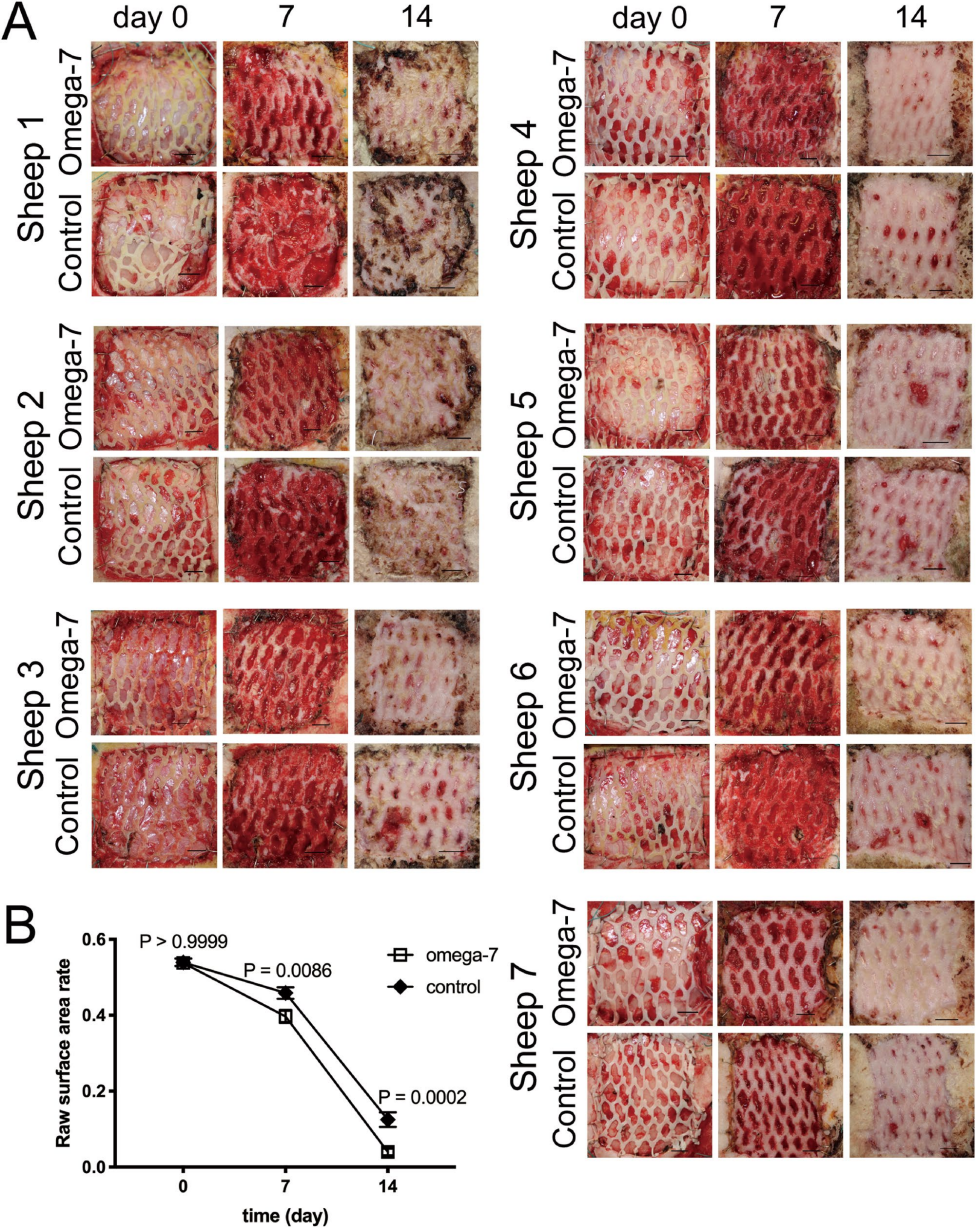
Effects of Omega-7 on ovine grafted burn wound healing. (**A**) Pictures of control and Omega-7-treated wounds in individual sheep at 0, 7 and 14 days. (**B**) Wound un-epithelialized surface area was measured by ultrasound and semi-quantified by Image J software. The raw surface area was measured in seven sheep. Each sheep had two treated and two untreated sites. Open circles indicate raw surface area in Omega-7, whereas closed circles indicate raw surface area in control sites. Data are expressed as mean ± SEM. Photographs taken and compiled, by authors, in Adobe Photoshop CC 2020 (https://www.adobe.com/jp/products/photoshop.html) without changing the content of images themselves.

All grafted sites had no complications such as infection or hematoma. On the day of the surgery, the sizes of the un-epithelialized raw surface areas of the grafted sites were comparable in the Omega-7 (0.54 ± 0.01) and control (0.54 ± 0.01) groups (P > 0.999). At post operation days (POD) 7 and 14, the size of the un-epithelialized raw surface area in the Omega-7 group (0.40 ± 0.02, and 0.04 ± 0.01, respectively) was significantly less than those of the control group (0.46 ± 0.02 [P = 0.0086] and 0.13 ± 0.02 [P = 0.0002], respectively) (Fig. 3).

**Figure 4:**
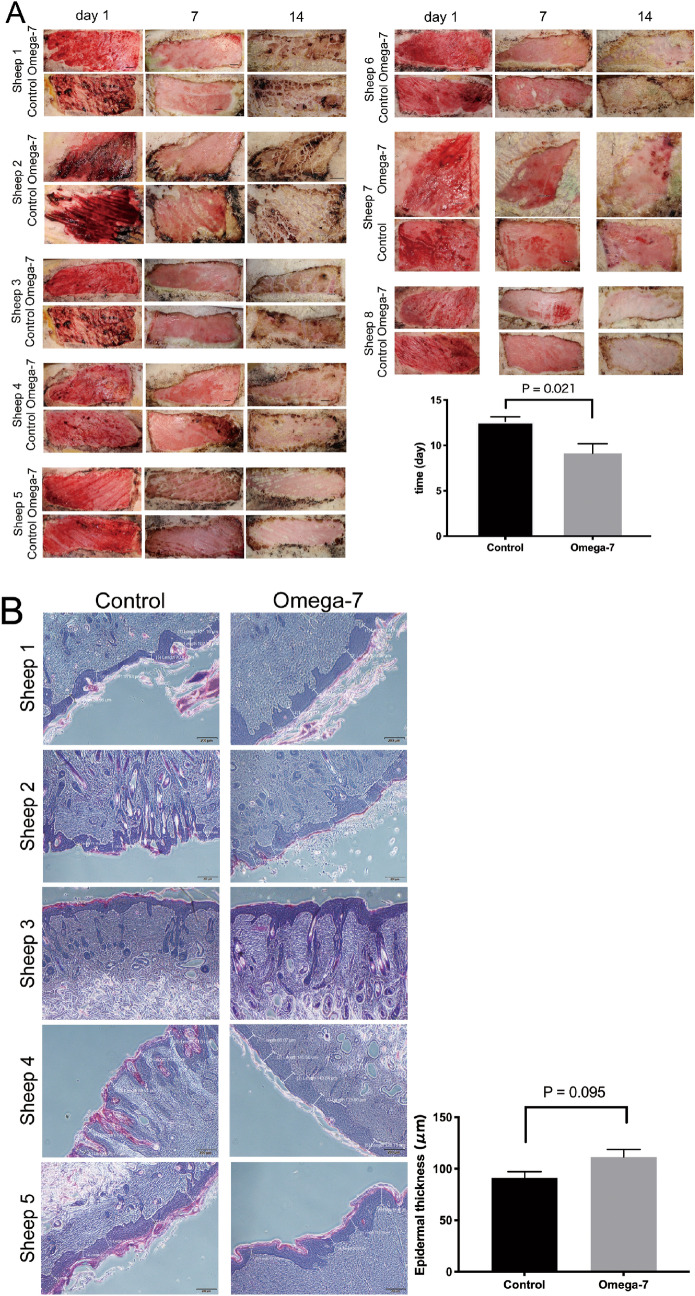
Day of complete epithelialization and epithelial thickness of donor sites. (**A**) The complete epithelialization of the donor sites was assessed by ultrasound examination. The assay was performed in eight sheep (8 treated and 8 control sites). The graph shows the complete epithelialization time (days) in donor sites. (**B**) In five sheep, the re-epithelialized epidermal thickness of donor sites was measured in five-places in each wound site. Data are expressed as mean ± SEM. Scale bar in the macroscopic image (**A**) is 5 mm and in the micrograph (**B**) is 200 μm. Photographs taken and compiled, by authors, in Adobe Photoshop CC 2020 (https://www.adobe.com/jp/products/photoshop.html) without changing the content of images themselves.

Donor sites had no complications such as infection, bleeding or ulceration. The complete epithelialization time in donor sites treated with Omega-7 (9.1 ± 1.0 days) was significantly shorter than of the control group (12.6 ± 0.5 days, P = 0.021) (Fig. 4A).

### Re-epithelialized epidermal thickness of donor sites in vivo

The re-epithelialized epidermal thickness of donor sites in Omega-7 group (111.4 ± 7.4 μm) was slightly higher than in control (91.2 ± 6.0 μm) group at POD14; however, no significant difference was found between the groups (P = 0.095, Fig. 4B).

### Blood flow of grafted and donor sites in vivo

**Figure 5:**
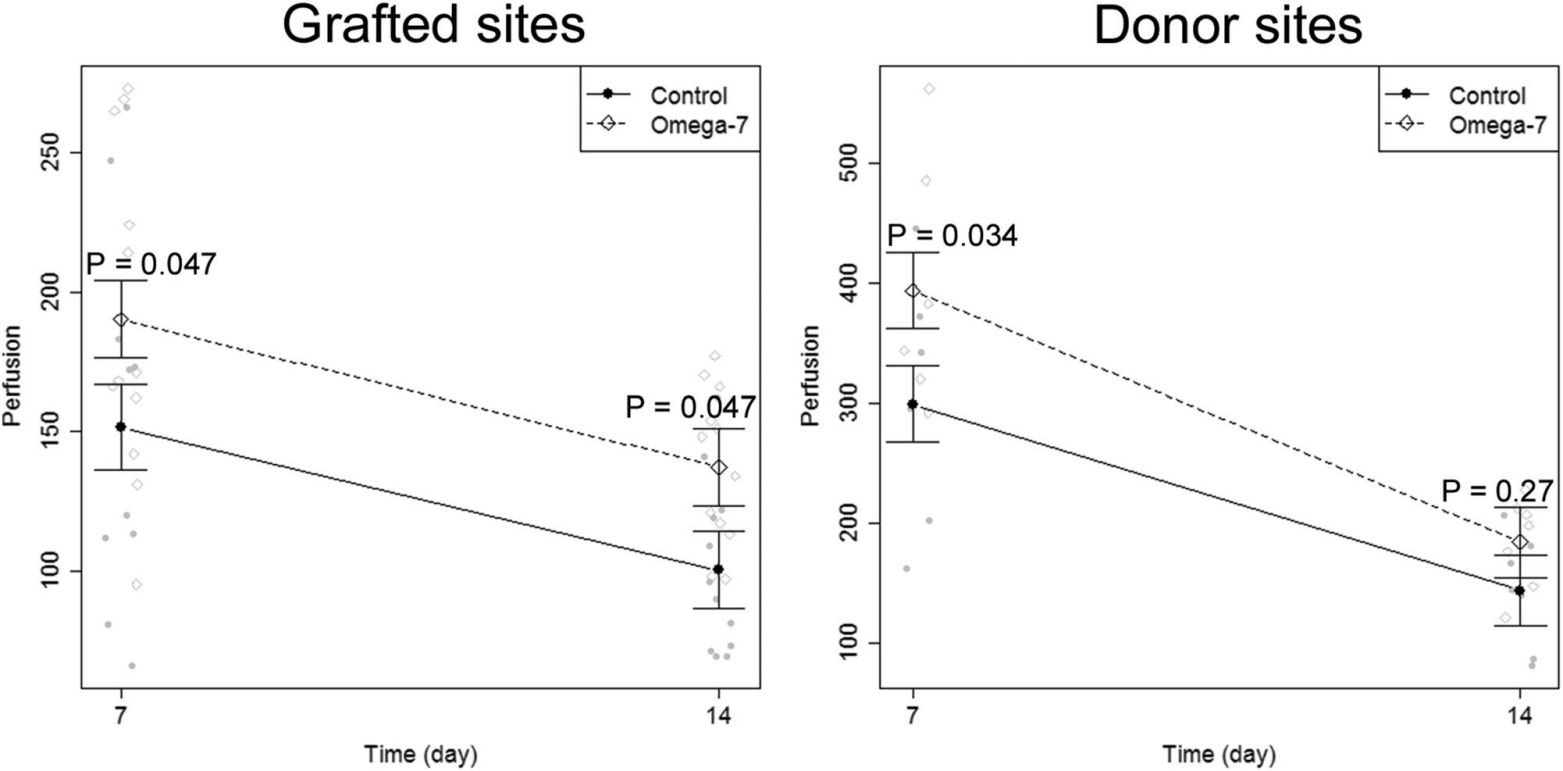
Blood flow of grafted burn and donor sites at 7 and 14 days. Blood flow was measured in six sheep (12 treated and 12 control sites) at grafted sites, and in seven sheep (7 treated and 7 control sites) at donor sites. Left panel: Blood flow in grafted burn wound sites. Right panel: Blood flow in donor sites. Data are expressed as mean ± SEM.

Table 1 illustrates the wound blood flow of each individual sheep. In summary, the blood flow, in the grafted sites, was significantly higher in the Omega-7 group at POD7 and POD14 (190.0 ± 16.9, and 137.2 ± 8.0 Perfusion Units [PU], respectively) than that of the control group (153.3 ± 21.2, and 100.2 ± 8.9 PU, respectively) (P = 0.047) (Fig. 5, left panel). In the donor sites, the blood flow in the Omega-7 group at POD7 (398.0 ± 43.0 PU) was significantly greater than that of the control group (303.3 ± 43.5 PU) (P = 0.034). However, the blood flow at POD14 was comparable between the Omega-7 (184.1 ± 14.5 PU) and control (143.6 ± 17.7 PU) groups (Fig. 5, right panel).

**Table 1:** Blood flow of grafted burn and donor sites at 7 and 14 days. Data are shown as mean ± SEM.

Sheep #	1	2	3	4	5	6	7
**Grafted site 7 days**							
Control			96.25 ± 15.75	219 ± 47	151.5 ± 31.5	89.5 ± 23.5	210 ± 37
Omega7		148.5 ± 17.5	128.5 ± 33.5	207.5 ± 65.5	239.5 ± 25.5	169.5 ± 1.5	246.5 ± 22.5
**Grafted site; 14 days**							
Control		71 ± 2.0	107.5 ± 11.5	85.5 ± 4.5	142 ± 20	125 ± 16	70 ± 1.0
Omega7		115 ± 2.0	145.5 ± 24.5	160 ± 6.0	155.5 ± 21.5	149.5 ± 1.5	97.5 ± 0.5
**Donor site; 7 days**							
Control		162	372	202	296	446	342
Omega7		344	563	383	292	320	486
**Donor site; 14 days**							
Control	81	86	166	140	181	207	144
Omega7	121	176	228	198	212	207	147

### Number of blood vessels in grafted skin burn wounds at POD14 in vivo

**Figure 6:**
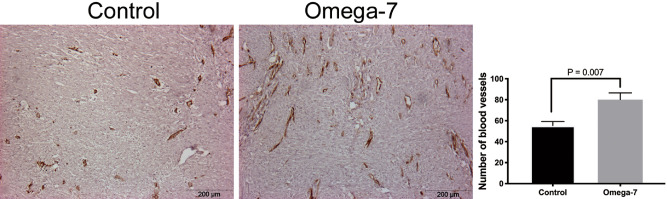
Number of blood vessels in grafted wounds. The number of vessels was counted using MMP-2 staining in grafted wounds at 14 days in eight sheep (8 treated and 8 control sites). Data are expressed as mean ± SEM. Scale bar in micrographs: 200 μm. Photographs taken and compiled, by authors, in Adobe Photoshop CC 2020 (https://www.adobe.com/jp/products/photoshop.html) without changing the content of images themselves.

Raw data on number of blood vessels in each individual sheep are shown in Table 2. In summary, the number of wound blood vessels semi-quantified using MMP-2 staining was significantly higher in the Omega-7 group (80.1 ± 6.4) than those in the control group (55.3 ± 3.9, P = 0.007) (Fig. 6).

**Table 2:** Number of blood vessels in grafted wounds at 14 days. Data are shown as mean ± SEM.

	Sheep 1	Sheep 2	Sheep 3	Sheep 4	Sheep 5	Sheep 6	Sheep 7
Control	49 ± 1.6	74 ± 7.9	54 ± 5.6	57 ± 13.6	61.4 ± 1.0	43 ± 8.5	48 ± 7.1
Omega7	66 ± 8.5	98.8 ± 11.8	70.4 ± 5.3	105.2 ± 8.5	87.8 ± 8.3	63.6 ± 9.1	70.4 ± 8.0

### KGF, 3-nitrotyrosine, and telomerase activity levels in grafted skin tissue at POD14

**Figure 7:**
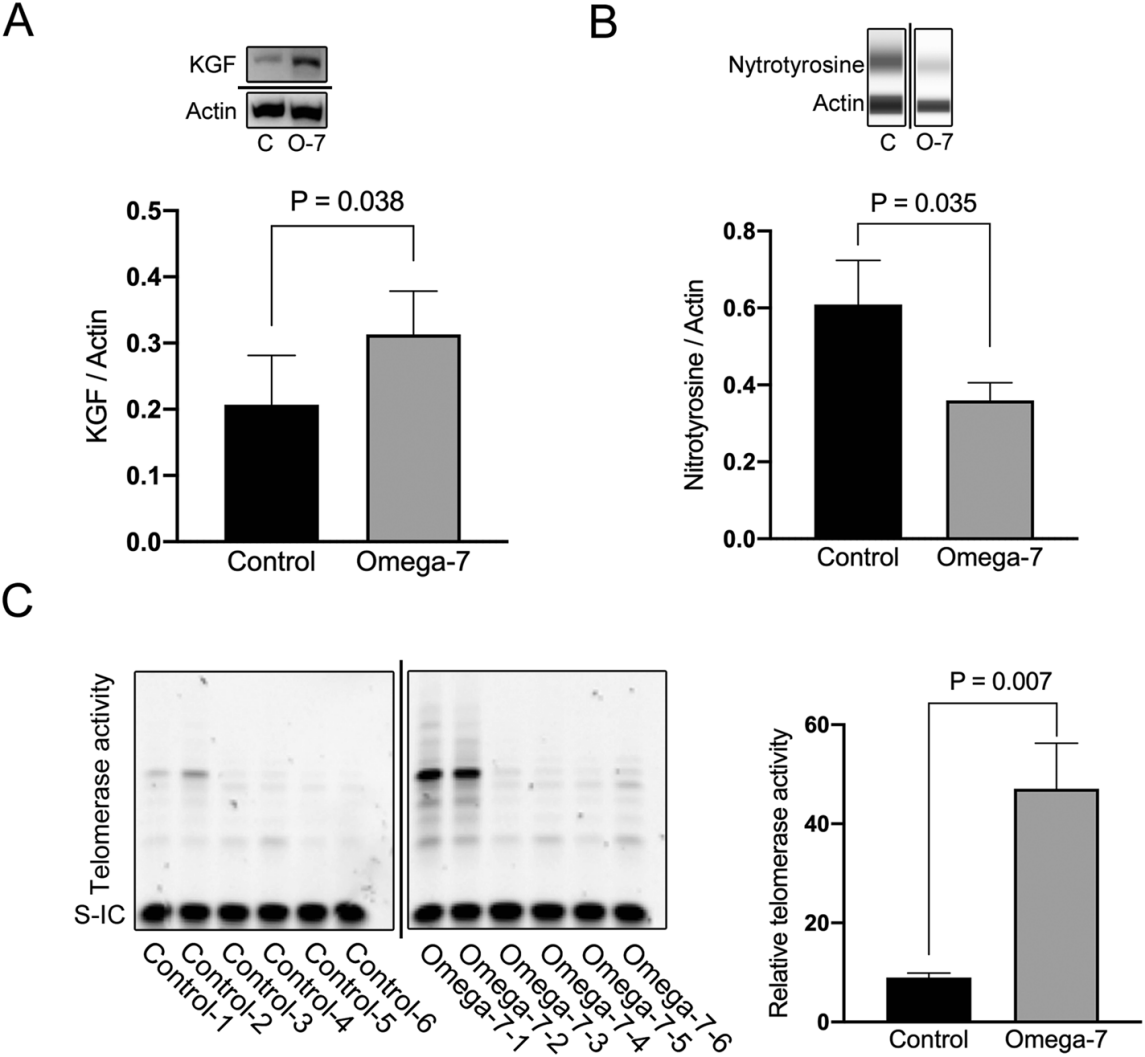
Keratinocyte growth factor (KGF), nitrotyrosine, and telomerase activity in grafted burn wounds at 14 days. (**A**) KGF were measured in four sheep (4 treated and 4 control sites). The level of KGF in skin tissue was significantly higher in the treatment group compared to the control group (0.21 ± 0.07 vs. 0.31 ± 0.07 relative unit). Full-length blots/gels are presented in Supplementary Fig. 1. (**B**) Nitrotyrosine were measured in eight sheep (8 treated and 8 control sites). The level of nitrotyrosine in skin tissue was significantly reduced in the treatment group compared to the control group (0.36 ± 0.05 vs. 0.61 ± 0.11 relative unit). Full-length blots/gels are presented in Supplementary Fig. 2. (**C**) Telomerase activity were measured in five sheep (6 Treated and 6 control sites). Telomerase activity measured by Telomeric Repeat Amplification Protocol (TRAP) and Gel-Based Telomerase Detection at 14 days after skin graft. The telomerase activity was significantly higher in Omega-7 grafted burn sites than that of control sites (47.1 ± 9.2 vs. 9.0 ± 0.9). The pictures show the telomerase activity of non-heat-treated samples in each group. Full-length blots/gels are presented in Supplementary Fig. 3. S-IC: signal from the internal standard in non-heat-treated samples. Data is expressed as mean ± SEM. Photographs taken and compiled, by authors, in Adobe Photoshop CC 2020 (https://www.adobe.com/jp/products/photoshop.html) without changing the content of images themselves.

The level of KGF in skin tissue was significantly higher in the treatment group compared to the control group (0.31 ± 0.07 vs. 0.21 ± 0.07 relative unit, P = 0.038) (Fig. 7A). The level of 3-nitrotyrosine in skin tissue was significantly reduced in the treatment group compared to the control group (0.36 ± 0.05 vs. 0.61 ± 0.11 relative unit, P = 0.035) (Fig. 7B). The telomerase activity was significantly higher in Omega-7-treated grafted burn sites than in control sites (47.1 ± 9.2 vs. 9.0 ± 0.9, P = 0.007) (Fig. 7C).

## Discussion

In the present study, we have tested the efficacy of Omega-7 unsaturated oils isolated from natural Sea buckthorn pulp oil in a clinical relevant ovine model of grafted burn wound healing.

The key findings of the present study are as follows: (1) Topical application of Omega-7 increased blood flow as well as the number of blood vessels and accelerated wound healing; (2) Omega-7 increased telomerase activity in grafted burn wounds in vivo; and (3) Omega-7 promoted keratinocyte proliferation (colony-forming rate) and migration (scratch assay) in vitro models of wound healing.

The importance of neovascularization for wound healing is well known^26^. Treatment with Omega-7 significantly increased the number of blood vessels and blood flow in the wound beds. This is consistent with findings by Upadhyay et al. who reported that sea buckthorn (SBT) oil containing abundant Omega-7 increased MMP-2 and MMP-9, which are known as important factors for neovascularization^4^ in rat burn wound models^24^. We have previously shown that SBT oil increases blood flow in ovine grafted burn wounds^21^, which was supported by findings by Seven et al. in rat burn models^27^.

To understand mechanisms of increased blood flow as well as neovascularization, we have measured KGF, also known as fibroblast growth factor (FGF)-7 and found it significantly increased by treatment with Omega-7. Niu et al. reported that KGF is important during the later stages of neovascularization when luminal spaces and basement membranes are being developed. KGF is known as a potent mitogen for vascular endothelial cells, and stimulates endothelial cells to produce a protease of urokinase-type plasminogen activator, which acts on neovascularization^28^. In our present study, although the direct link remains unknown, Omega-7 may have increased blood flow via increasing KGF.

On the other hand, cell proliferation and migration is important for wound re-epithelialization. Our in vitro study results show that Omega-7 treatment accelerated keratinocyte colony-forming rate and improved their migration. Various cytokines have been reported to promote keratinocyte migration^29^. Among them, KGF plays an important role for re-epithelialization by stimulating proliferation and migration of keratinocytes^29^. As mentioned, the topical application of Omega-7 increased wound KGF, suggesting that Omega-7 may have also increased keratinocyte proliferation and migration, and accelerated wound re-epithelialization via increasing KGF.

In the present study, we also demonstrated that topical application of Omega-7 upregulated telomerase activity in grafted wound keratinocytes. Counter et al. reported that the epidermis telomere length, after cultured keratinocytes sheet grafting in burn patients, is shortened compared to those of normal epidermis in the same patient^30^. This may be one of the mechanisms leading to long term complications such as graft fragility or blister formation. Buckingham et al. indicated that telomerase is activated in the epidermis as it is needed for cell proliferation and damage repair^31^. Taken together, the results of the present and previous studies suggest that the topical application of Omega-7 may have promoted keratinocyte proliferation via increasing telomerase activity and accelerating wound re-epithelialization.

It should be noted that oxidative damage is a major cause determining the rate of loss of telomeric DNA and telomere shortening^12^. Parihar et al. reported increased oxidative stress in burn patients^32^. It is known that SBT reduces oxidative stress in blood cells and some organs^33,34^. In the present study, we report that Omega-7 significantly reduced the wound bed 3-nitrotyrisine. In addition, KGF is known to increase the transcription of factors involved in the detoxification of oxidative stress^35^. In brief, previous studies strongly suggest a possible involvement of reduced oxidative stress, KGF and telomorese activity in burn wound healing. Although, we were not able report causative effects of those factors, we speculate that Omega-7 may have improved wound healing via inhibiting oxidative stress and promoting KGF and telomerase activity. Further studies should focus on interlink between these factors.

Even though the current study shed some valuable insights into the role of Omega-7 in wound healing, there are a few limitations: (1) We did not directly measure the telomere size; (2) Although we have shown that Omega-7 attenuates 3-nitrotyrosine levels, precise mechanisms by which Omega-7 increased KGF and telomerase activity remain unknown. Additionally, we did not investigate exact mechanisms of how telomerase activity promoted keratinocyte proliferation and migration. As mentioned, the present study did not show a direct link between telomere size and telomerase activity. Sarin et al. showed that telomerase promoted cell proliferation of quiescent, resting multipotent stem cells in the hair follicle bulge region through a non-canonical pathway^36^. Thus, we do not exclude the possibility that Omega-7-induced telomerase activity may have promoted cell proliferation and migration by multiple mechanisms; (3) No studies have been performed to explore a possible direct link between KGF and telomerase activity; (4) Because of limited availability of the reagents/kits, KGF and telomerase activity have not been measured in all 8 sheep; (5) In the present study, we were not able to measure half-life time of Omega-7 (previously, it was shown to be 9 h^37^). We have also not performed its inhibition assay. Further studies are warranted to investigate these underlying mechanistic aspects, and perform cytotoxicity assays to reveal possible side effects; and (6) Finally, present study did not consider possible impact of wound dressing on the healing process. Previous studies reported potential impacts of various wound dressing^38–41^. Zhao et al. demonstrated that nanocomposite cryogels as injectable shape memory hemostatic dressings not only promoted the wound healing process but also exerted hemostatic effects compared to the gauze dressing^38^. El Fawal e al. reported antimicrobial activities of novel wound dressing with hydroxyethyl cellulose-based hydrogel membranes^39^.

Nevertheless, taken together, the results of present and previous studies support our notion that Omega-7 significantly increases the wound bed cell telomerase activity and KGF, and accelerates grafted burn wound healing, possibly by inhibiting burn-induced oxidative stress. Sheep are frequently used to mimic clinical scenarios of human disease and treatment because the anatomy of their organs (e.g. skin and subcutaneous tissue, lung, and nerves) are similar to those in humans^42–47^. Therefore, we believe that our findings in this study are highly translational and capable of being imported into clinical practice, as the clinically relevant ovine model resembles all aspects of wound healing in burn patients, i.e., escharectomy within 24 h after burn, pressure bandage coverage of grafted wounds for a week, daily wound washing and changing of dressings, daily wound closure assessment, and continuous hemodynamic monitoring and resuscitation in an ICU setting.

## Conclusions

Topical application of Omega-7 accelerates healing of grated burn wounds in ovine model. Further studies are warranted to explore mechanistic aspects underlying its salutary effects.

## Materials and methods

### Preparation of Omega-7

Omega-7 was isolated from sea buckthorn pulp oil (Polyvit Co., Ltd., Gangar Holding, Ulaanbaatar, Mongolia) using Florisil column chromatography method. Then, it was dissolved in 100% ethanol and 0.1% v/v DMSO. The pH was adjusted to 5.22 with NaOH. For in vitro studies, concentrations of 0.025% and 0.08% were used for colony counting and migration assays, respectively. These doses were chosen based on initial toxicity studies. For colony counting, we have evaluated effects of Omega-7 in concentration ranges of 0.025% and 0.08% and 0.025% was chosen as an optimal dose because the colonies were not formed with dose of 0.08% (Fig. 1B). For the migration assay, we assessed concentration ranges of 0.08% to 0.4% and found that 0.08% was the most effective dose. Concentrations exceeding 0.1% negatively affected the cells (Fig. 2B). For in vivo studies, 4% Omega-7 diluted in 0.9% normal saline (0.1 mL/cm^2^) was used.

### Isolation and culture of ovine keratinocytes

Ovine hair was removed by using 3-min hair removal cream. Skin was thoroughly washed before the harvesting. Then, the epidermis with 0.007 inches thickness was taken by dermatome and washed twice under sterile conditions with PBS 1X containing 200U/ml penicillin and 200 μg/ml streptomycin (Gibco by Life Technologies) and trimmed into 0.2 × 0.2 cm pieces. Then, it was digested in 0.25% trypsin-2.21 mM EDTA (Corning) solution at 37 °C and 15×*g* for 30 min in a processing unit from Ingeneron Incorporated. The trypsinized tissue was strongly shacked and left rest for few minutes. The supernatant was transferred to another tube (KC1) through a cell strainer of 70 µm where the digestion was stopped by addition of equal volume of DMEM high-glucose medium (Gibco by Life Technologies) that contained 10% fetal bovine serum (FBS) (Corning), 100U/ml of penicillin and 100 μg/ml streptomycin, relative to the volume of trypsin. Then, keratinocyte isolation tube was filled up with 0.25% trypsin-2.21 mM EDTA (Corning) solution and returned to the processing unit for another 30 min. The suspension (KC1) was centrifuged at 200×*g* for 5 min, the supernatant was discarded, and the cell pellet was re-suspended in complete keratinocyte growth medium. The cells were kept on the incubator (5% CO_2_) at 37˚C until the tissue processing is completed.

### Keratinocyte colony counting (proliferation) assay

Keratinocytes in passage 2 were seeded (24,000 cells/cm^2^) in either complete growth medium containing 0.1% DMSO (control group); or complete growth medium with 0.025% Omega-7 dissolved in 0.1% DMSO (treatment group) onto a fresh feeder layer of mitomycin C-treated 3T3 fibroblasts^48^ that had been prepared 24 h earlier at the same cell density as the keratinocytes in 6 well plates. The cells were cultured for 96 h at the same conditions described above. The number of colonies in 5 fields per well were counted at 72 and 96 h after treatment.

### Keratinocyte migration (scratch) assay

Confluent monolayers of ovine keratinocytes in passage 2 were scratched using a 100 μl pipet tip on a 6 well plate. Keratinocyte medium containing 0.1% DMSO with (Omega-7 group; n = 4) or without (Control group; n = 4) 0.08% Omega-7 were added to the plate. Just after scratch (t = 0) and 17 h after scratch (t = 17), 5 to 8 digital photographs per well were taken. The wound area at each time point (i.e., A_t=0_ and A_t=17_) was calculated using ImageJ software version 1.50 (National Institutes of Health, Bethesda, MD). The wound closure percentage was calculated by the following equation:

$$ {\text{Percent }}\;{\text{of }}\;{\text{wound }}\;{\text{closure }}\left( \% \right) = \frac{{{\text{At}} = 0{ } - {\text{ At}} = 17}}{{{\text{At}} = 0{ }}} \times 100 $$

### Animal care

The Institutional Animal Care and Use Committee (IACUC) at the University of Texas Medical Branch approved this study, including the number of animals used. The guidelines of the National Institutes of Health for the care and use of experimental animals were carefully followed. Animals were individually housed in metabolic cages. Burn injury and skin grafting procedures were performed under deep isoflurane anesthesia and buprenorphine analgesia. After these procedures, sheep were studied in a conscious state for 14 days. Post-surgical analgesia was provided by long acting buprenorphine.

### Surgical preparation and experimental protocol

Eight female Merino sheep (31.5 ± 4.5 kg, approximately 3 years old; Talley Ranch, Bastrop, TX) were used in this study. The animals were chronically instrumented for hemodynamic monitoring with a right femoral artery catheter and a 7-French Swan-Ganz thermodilution catheter under aseptic conditions. Two full-thickness flame burn sites of 25 cm^2^ were made on both sides of dorsum with 2 cm distance between sites and 3 cm apart from the spine under anesthesia and analgesia. For the induction of burn sites, we have used a metal sheet with 25 cm^2^ open space in the middle. Underneath the metal sheet, a burn resistant cloth was instilled to prevent the heat to the adjacent tissues. The burn was induced by fire flame, which was applied approximately 3 s until the skin shrinkage stops. The method was previously established in our laboratory that the full-thickness skin burn was confirmed by the histological analysis^49^.

**Figure 8:**
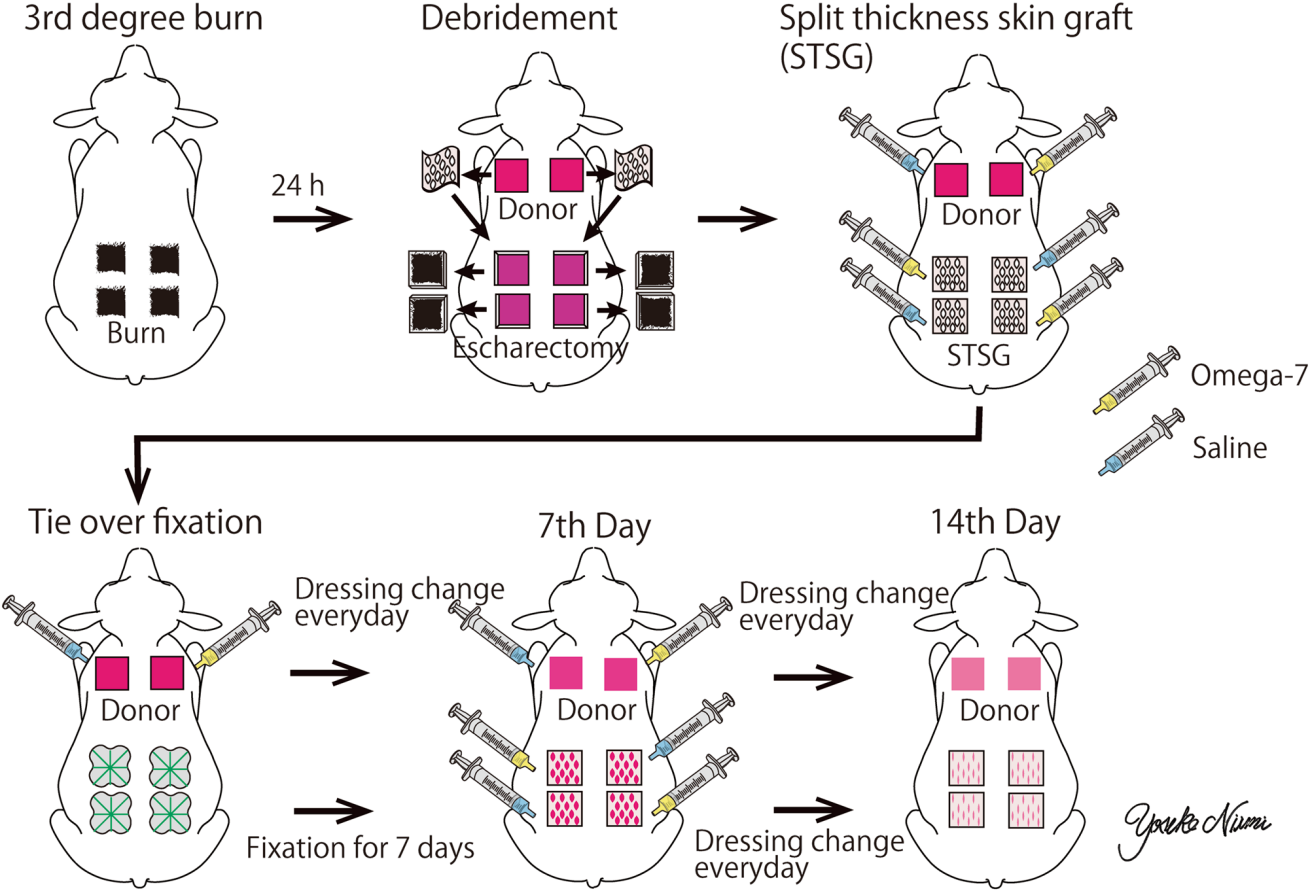
Experimental design of clinically relevant ovine grafted burn wound healing model. The third-degree burn was induced by fire flame (above left). 24 h after the burn, the eschar was excised down to the fascia. Split-thickness skin grafts 0.03 inches thick were harvested from sites remote from the burned sites to be meshed and grafted to the wounds (above center). 0.1 ml/cm^2^ of 4% Omega-7 in 0.9% saline was topically applied to one of two randomly selected autografted sites (Omega-7 group). The remaining site received the same amount of 0.9% saline as a control (control group). The donor sites were also randomly allocated to 0.1 ml/cm^2^ of 4% Omega-7 or 0.9% saline (above right). After the initial treatment, the grafted sites were covered with tie-over dressings removed at postoperative day (POD) 7 (below left). At the 7 days after the grafting, the wounds were gently washed daily with a sterile saline without anesthesia (below center). Thereafter, the wounds were treated with Omega-7 or 0.9% saline, and covered with polyurethane foam for 7 days (14 days total duration of experiments). The donor site wounds were covered with polyurethane film dressing and treated daily with 4.0% Omega-7 or 0.9% saline starting at POD1 for 14 days (below right). The study design was drawn using Adobe illustrator by the first author (https://www.adobe.com/jp/products/illustrator.html).

Twenty-four hours after the burn, the eschar was excised down to the fascia. Split-thickness skin grafts 0.03 inches thick were harvested from sites remote from the burned sites to be meshed and grafted to the wounds. 0.1 ml/cm^2^ of 4% Omega-7 in 0.9% saline was topically applied to one of two randomly selected autografted sites (Omega-7 group). The remaining site received the same amount of 0.9% saline as a control (control group). The donor sites were also randomly allocated to 0.1 ml/cm^2^ of 4% Omega-7 or 0.9% saline. After the initial treatment, the grafted sites were covered with tie-over dressings removed at postoperative day (POD) 7. At the 7 days after the grafting, the wounds were gently washed daily with a sterile saline without anesthesia. Thereafter, the wounds were treated with Omega-7 or 0.9% saline, and covered with polyurethane foam for 7 days (total duration of the study is 14 days) (Fig. 8). No wound debridement was done.

The donor site wounds were covered with polyurethane film dressing (Tegaderm, 3 M, MN) and treated daily with 4.0% Omega-7 or 0.9% saline starting at POD1. Fourteen days after surgery, animals were deeply anesthetized and euthanized by intravenous administration of xylazine (3.0 mg/kg), ketamine (40 mg/kg), and buprenorphine (0.01 mg/kg) following IACUC approved protocols and American Veterinary Medical Association Guidelines for Euthanasia.

### Planimetric wound healing assessments

After the initial autografting and during each dressing change, standardized digital photographs of the wound fields were taken. A calibrated benchmark was positioned adjacent to each wound when photographs were taken. Photographs were processed using ImageJ. In grafted sites, for each wound at POD 0, 7, and 14, the total wound area (A_T_) and open mesh interstices of autografts (un-epithelialized raw surface area, A_U_) were measured. The proportion of the total wound surface area that was un-epithelialized (A_R_) was calculated by the: A_R_ = A_U_/A_T_.

The time required for complete epithelialization of donor sites were also determined. Un-epithelialized area was identified by high resolution ultrasound and semiquantified using image J as described in our previous study^21^.

### Blood flow measurement in grafted and donor sites

The blood flow was measured in both treated and untreated autograft and donor sites of six sheep using a LASER Doppler device (PeriFlux System 5000 ModelPF5001, Perimed AB, Sweden) at POD 7 and 14. Two locations were randomly chosen in each site for blood flow measurements and the means were analyzed.

### Histology

Wound biopsies were taken at two different locations from each donor site. Four-micrometer-thick cross-sections through the wound center were stained with hematoxylin and eosin (HE) according to standard histologic procedures. Images were acquired with an Olympus CKX41 microscope. The thickness of the epithelial layer of the donor site in each group was measured using software (CellSens Standard 1.11, Olympus, Tokyo). In the images taken at a magnification of 400, five measurements per field (total four fields per sheep) were acquired by an independent masked pathologist.

### Quantification of blood vessels

Immunolocalization of matrix metallopeptidase 2 (MMP-2), as a well-known marker for the angiogenesis^50–52^, was determined for semi-quantification of blood vessels using 1:200 diluted monoclonal antibody against human MMP-2 antigen (MS-806-P1, Thermo Fisher Scientific, MI) following previously established protocol^53^. Five images per slide were taken at a magnification of 100, and the number of MMP-2-stained vessels in each area was counted by a masked pathologist using Image J.

### Western blot analysis

We measured 3-nitrotyrosine and keratinocyte growth factors (KGF) in grafted skin tissue at POD14 in each group. The 3-nitrotyrosine was measured using an automated capillary Western blot analyzer (Wes, ProteinSimple, CA) system (n = 8). KGF was measured by a conventional Western blot method (n = 4). The skin tissue was homogenized, lysed, and the protein levels were measured using anti-3-nitrotyrosine antibody (06-284; MilliporeSigma, MA) and anti-KGF antibody (ab131162; Abcam, MA).

### Telomerase activity

Telomerase activity was measured in grafted skin tissue at POD14 in each group. Skin tissue (30 mg) was homogenized as described by Smith et al.^54^. All sample extracts were evaluated for heat sensitivity by incubation at 85 °C for 30 min. The telomeric repeat amplification protocol (TRAP) assay was performed according to the TRAPeze kit (S7700, EMD Millipore, MA, USA). 100 ng/μl of each sample and 2 units of DNA Polymerase (Nova Taq, Millipore-Novagen) were used per reaction. The following parameters were measured: (1) primer-dimer/PCR contamination control (r_0_); (2) telomerase quantitation control template (r); (3) the signal of the region of the gel corresponding to the TRAP product ladder bands in non-heat-treated (${\mathrm{x}}$); (4) heat-treated sample extracts ($\mathrm{x}$_0_); (5) the signal from the internal standard in non-heat-treated samples ($\mathrm{c}$); and (6) the telomerase quantitation control template ($\mathrm{cR}$). The total product generated (TPG) was calculated by the following equation:

$$ {\text{TPG}} = \frac{{\left( {{\text{x}} - {\text{x}}0} \right)/{\text{c}}}}{{\left( {{\text{r}} - {\text{r}}0} \right)/{\text{cR}}}} \times 100 $$

### Statistical analysis

Values are expressed as means ± SEM. The un-epithelialized area and blood flow were modeled with relation to the treatment group and time by mixed effects ANOVA. Differences between groups at each time point were estimated by Hommel-adjusted contrasts using R statistical software^55^. Other analyses were performed by paired t-test with GraphPad Prism version 7.0c for Mac OS X (GraphPad Software, CA). The significance level was set at P < 0.05.

## Supplementary Information

Supplementary Information.
