# Supplementary material for: Omega-7 oil increases telomerase activity and accelerates healing of grafted burn and donor site wounds
Source: Sci Rep. 2021 Jan 13;11:975. doi: 10.1038/s41598-020-79597-0 (PMC7806965; doi:10.1038/s41598-020-79597-0)
Supplement: Supplementary file 1 — Supplementary Information. [file 41598_2020_79597_MOESM1_ESM.docx]

**Title Page**

**“Omega-7 Oil Increases Telomerase Activity and Accelerates Healing of Grafted Burn and Donor Site Wounds”**

**Authors List:** Yosuke **Niimi** MD, PhD^#1^, Dannelys **Pérez-Bello** PhD^#2^, Koji **Ihara** MD^3^, Satoshi **Fukuda** MD^4^, Sam **Jacob** MS^5^, Clark R. **Andersen** MS^6^, Tuvshintugs **Baljinnyam** PhD^7^, Jisoo **Kim** MD^8^, Suzan **Alharbi** MS^9^, Donald S. **Prough** MD^10^ and Perenlei **Enkhbaatar** MD, PhD^11^

# YN and DP contributed equally to this manuscript.

1. Yosuke Niimi MD, PhD. Department of Anesthesiology, University of Texas Medical Branch, 301 University Blvd, Galveston, Texas 77555-1102, USA. Department of Plastic and Reconstructive Surgery, Tokyo Women’s Medical University; 8-1, Kawada-cho, Shinjuku-ku, Tokyo.

E-mail address: niimiyousuke2000@gmail.com

2. Dannelys Pérez-Bello PhD. Department of Anesthesiology, University of Texas Medical Branch, 301 University Blvd, Galveston, Texas 77555-1102, USA.

E-mail address: dannebello@yahoo.com

3. Koji Ihara MD. Department of Plastic and Reconstructive Surgery, Tokyo Women’s Medical University; 8-1, Kawada-cho, Shinjuku-ku, Tokyo.

E-mail address: kojiihara@hotmail.com

4. Satoshi Fukuda MD. Department of Anesthesiology, University of Texas Medical Branch, 301 University Blvd, Galveston, Texas 77555-1102, USA.

E-mail address: safukuda@utmb.edu

5. Sam Jacob MS. Department of Pathology, Shriners Hospitals for Children, 815 Market St, Galveston, Texas, 77550, USA

E-mail address: sjacob@utmb.edu

6. Clark R. Andersen MS. Department of Biostatistics, University of Texas Medical Branch,

301 University Blvd, Galveston, TX 77555-1102, USA

E-mail address: clanders@utmb.edu

7. Tuvshintugs Baljinnyam PhD. Department of Anesthesiology, University of Texas Medical Branch, 301 University Blvd, Galveston, Texas 77555-1102, USA.

E-mail address: tuvshintugsd@gmail.com

8. Jisoo Kim MD. Department of Anesthesiology, University of Texas Medical Branch, 301 University Blvd, Galveston, Texas 77555-1102, USA

E-mail address: jisookim614@gmail.com

9. Suzan Alharbi MS. Department of Anesthesiology, University of Texas Medical Branch, 301 University Blvd, Galveston, Texas 77555-1102, USA

E-mail address: sualharb@utmb.edu

10. Donald S. Prough MD. Department of Anesthesiology, Department of Anesthesiology, University of Texas Medical Branch, 301 University Blvd, Galveston, Texas 77555-1102, USA.

E-mail address: dsprough@utmb.edu

11. Perenlei Enkhbaatar MD, PhD. Department of Anesthesiology, University of Texas Medical Branch 301 University Blvd, Galveston, Texas 77555-1102, USA.

E-mail address: peenkhba@utmb.edu

*Corresponding author*

Perenlei Enkhbaatar MD, PhD.

Department of Anesthesiology, University of Texas Medical Branch 301 University Blvd, Galveston, Texas 77555-1102, USA.

E-mail address: peenkhba@utmb.edu

Tel: (409) 747-0096
Fax: (409) 772-6409

**Supplementary Figure and Figure Legends**

Supplementary Figure 1


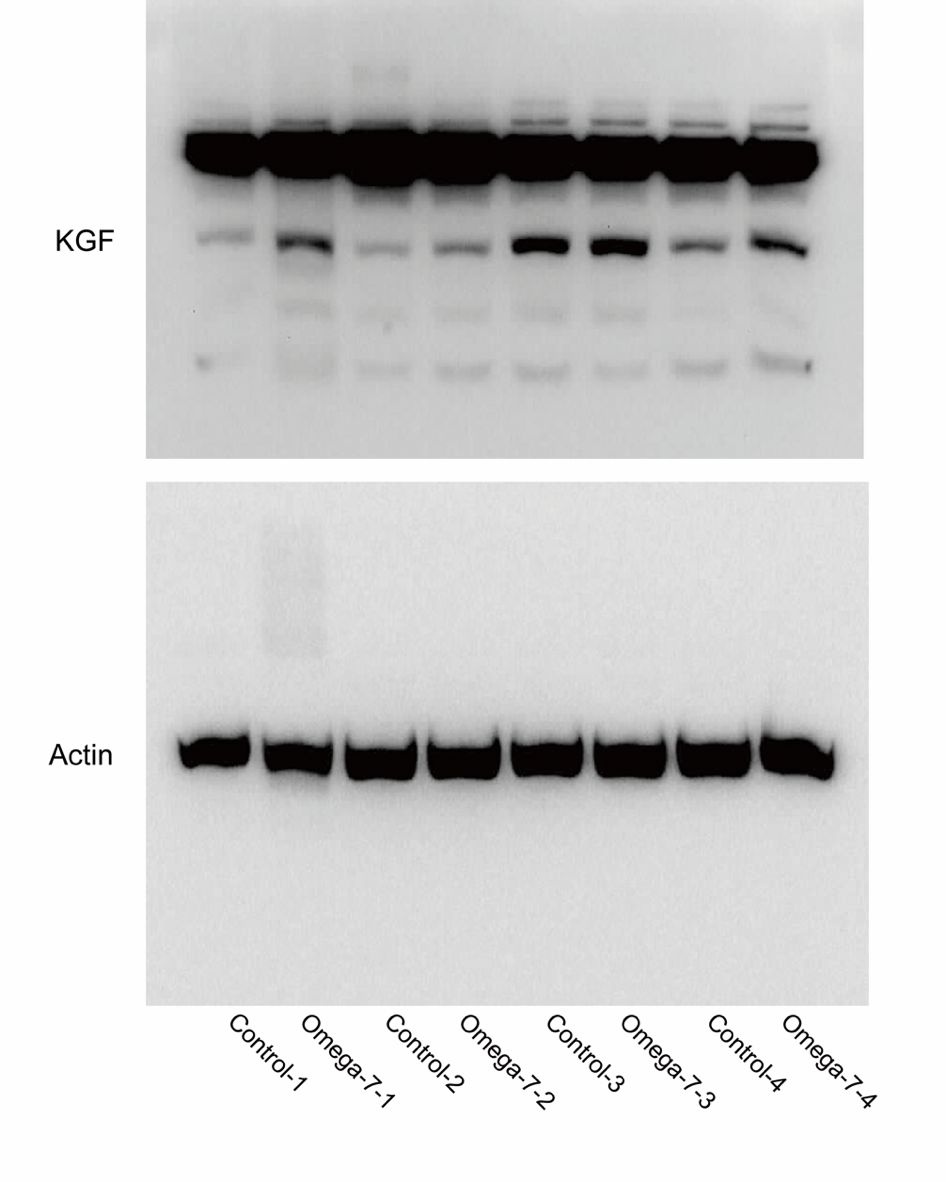


Keratinocyte growth factor (KGF) in grafted skin tissue at POD14 in each group were measured by a conventional Western blot method (n=4). The skin tissue was homogenized, lysed, and the protein levels were measured using anti-KGF antibody (ab131162; Abcam, MA). Photographs taken and compiled, by authors, in Adobe Photoshop CC 2020 (<https://www.adobe.com/jp/products/photoshop.html>) without changing the content of images themselves.

Supplementary Figure 2


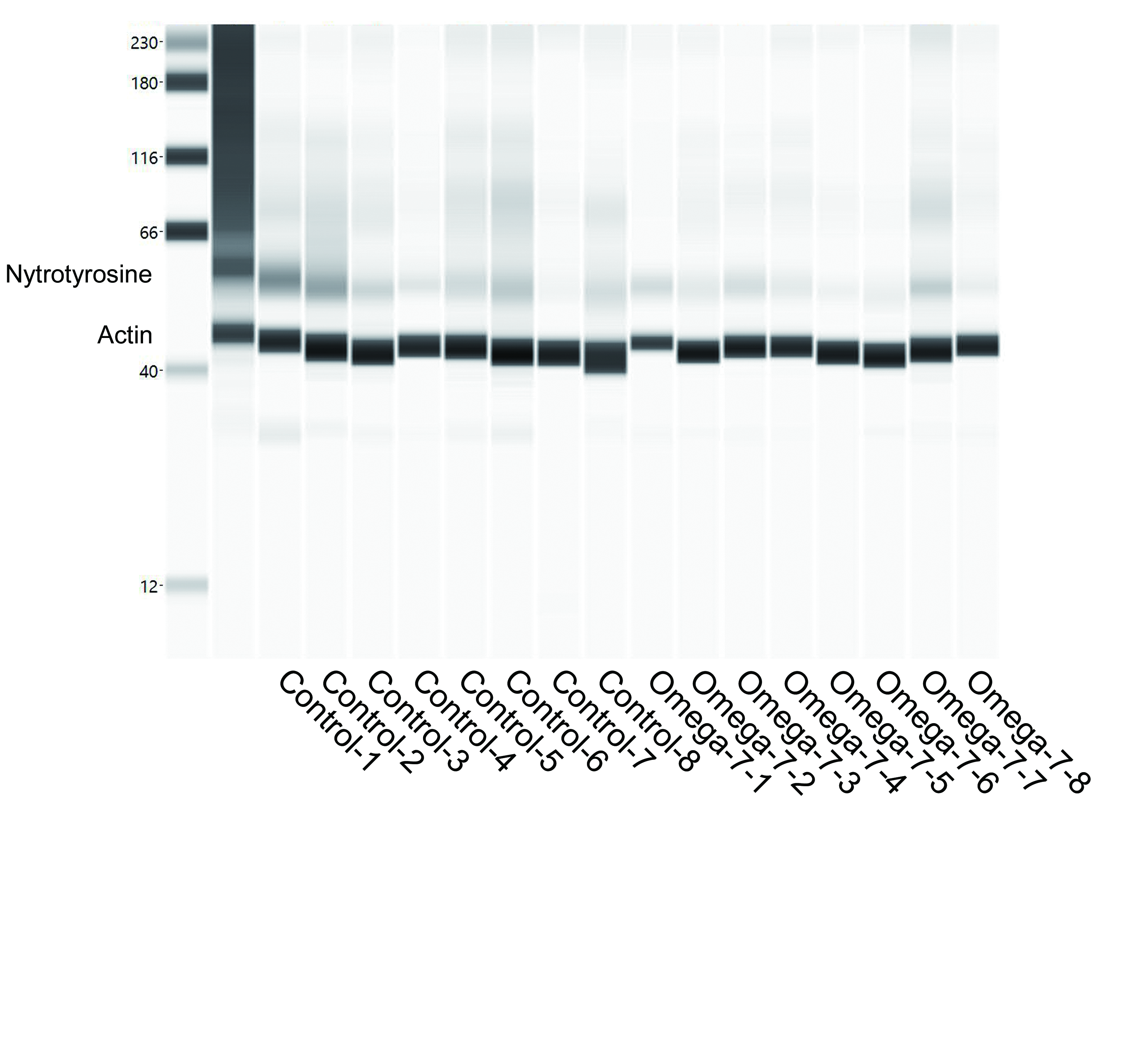


The 3-nitrotyrosine in grafted skin tissue at POD14 in each group were measured using an automated capillary Western blot analyzer (Wes, ProteinSimple, CA) system (n=8). The skin tissue was homogenized, lysed, and the protein levels were measured using anti-3-nitrotyrosine antibody (06-284; MilliporeSigma, MA).

Supplementary Figure 3


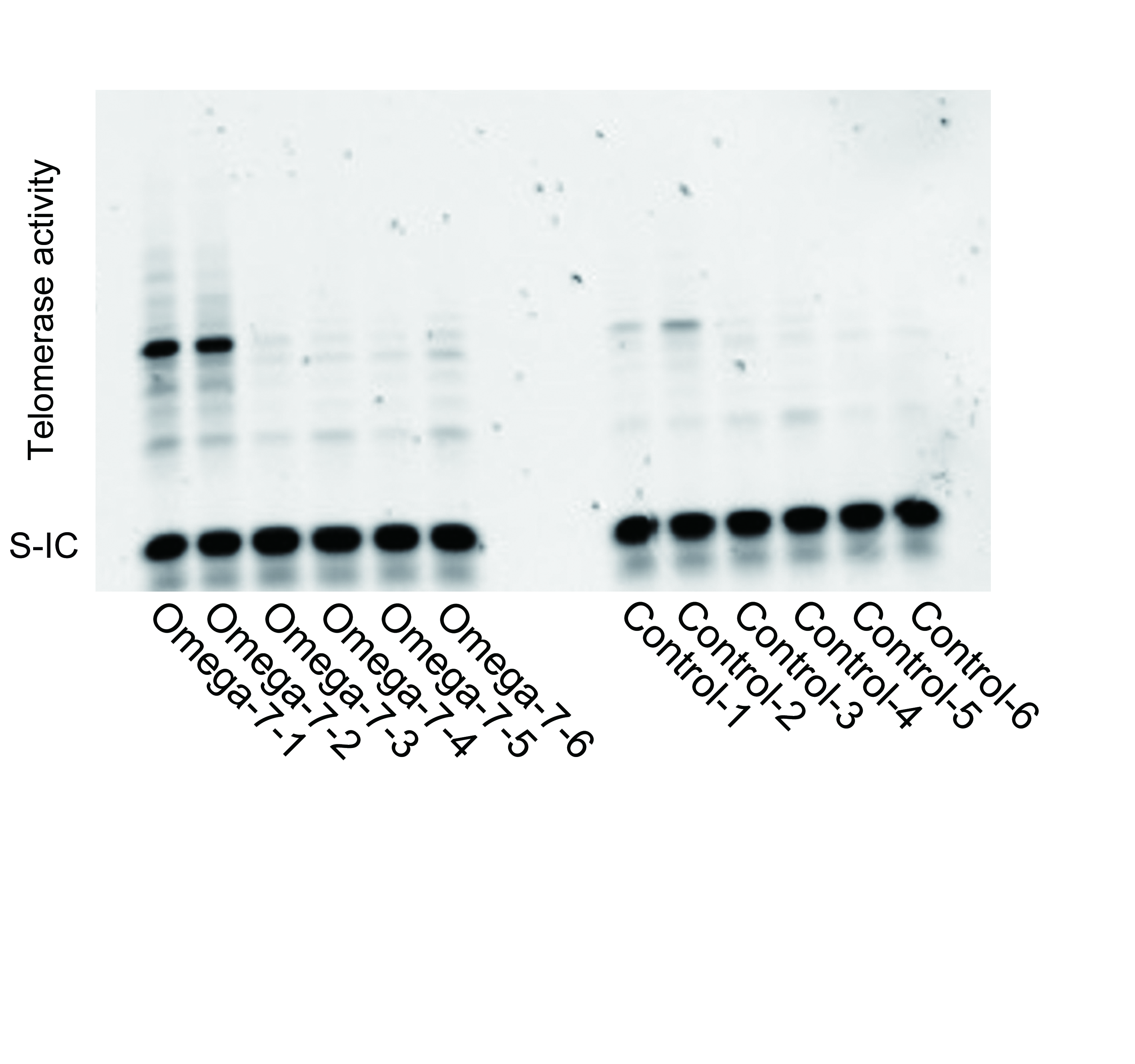
Telomerase activity in grafted skin tissue at POD14 in each group were measured by Telomeric Repeat Amplification Protocol and Gel-Based Telomerase Detection (n=6).

S-IC: signal from the internal standard in non-heat-treated samples.

Photographs taken and compiled, by authors, in Adobe Photoshop CC 2020 (<https://www.adobe.com/jp/products/photoshop.html>) without changing the content of images themselves.
